# The complete chloroplast genome of an endangered plant *Artemisia borotalensis* (Asteraceae) and phylogenetic analysis

**DOI:** 10.1080/23802359.2022.2163599

**Published:** 2023-01-15

**Authors:** Guang-Zhao Jin, Zhi-bin Wen, Feng Song, Ying Feng

**Affiliations:** a State Key Laboratory of Desert and Oasis Ecology, Xinjiang Institute of Ecology and Geography, Chinese Academy of Sciences, Urumqi, China; b The Herbarium of Xinjiang Institute of Ecology and Geography, Chinese Academy of Sciences, Urumqi, China; c University of Chinese Academy of Sciences, Beijing, China; d Key Laboratory of Plant Resources Conservation and Sustainable Utilization, South China Botanical Garden, Chinese Academy of Sciences, Guangzhou, China

**Keywords:** *Artemisia borotalensis*, Asteraceae, complete chloroplast genome, phylogenetic analysis

## Abstract

*Artemisia borotalensis* Poljakov is an endemic and endangered herb in China. In this study, we sequenced and analyzed the complete chloroplast genome of this species. Sequencing revealed the genome to be 151,179 bp in length, containing a large single copy region (82,862 bp), a small single copy region (18,377 bp), and a pair of inverted repeat regions (24,970 bp each). Our analyses demonstrated that it contained 133 genes, including 87 protein-coding genes, 37 transfer RNA genes, eight ribosomal RNA genes, and one pseudogene (*ycf1*). Furthermore, we found the genome to have an overall GC content of 37.4%. A phylogenetic analysis indicated that *A. borotalensis* and *A. maritima* clustered together as sister group to *A. annua* and *A. fukudo* clade.

## Introduction

**Figure 1.**
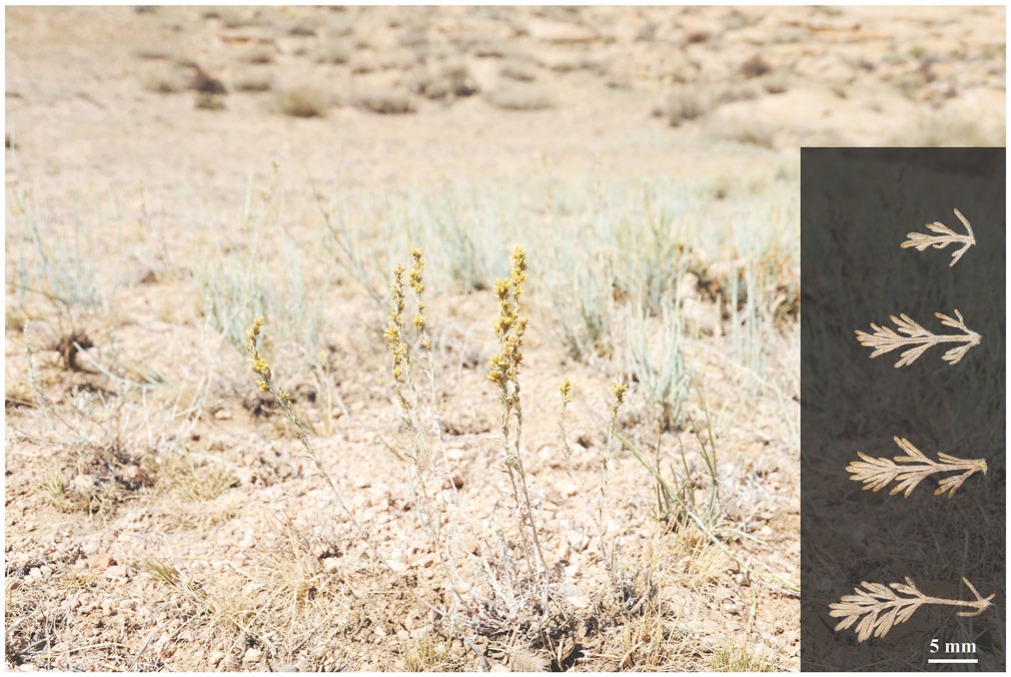
Field photograph of *Artemisia borotalensis* Poljakov. Main identifying traits: elliptic leaf blades, densely gray tomentose leaves that are 2-pinnatisect; three to five pairs of segmented leaves with linear lobules; synflorescence formed of narrow spike-like panicles.

*Artemisia borotalensis* Poljakov 1954, an endemic plant in China, is a perennial herb from the family Asteraceae (Ling et al. 2011). In China, *A. borotalensis* is distributed over desert or semi-desert grassland in the Gobi and on gravel hillside areas in Xinjiang (Figure 1). Most *Artemisia* species have important medicinal, ecological, and economic value (Duffy and Mutabingwa 2006; Vallès et al. 2011). Song et al. (2021) reported that the essential oils contained in *A. borotalensis* can effectively kill cotton aphids and psyllids. However, *A. borotalensis* has become an endangered species due to its relatively narrow area of distribution and the disturbance of its original habitat destroyed by human activities. Therefore, using the genome skimming approach, we constructed the complete chloroplast genome of *A. borotalensis*, which will contribute to the conservation of this taxon’s genetic resources and the understanding of its phylogenetic relationships.

## Materials

An *A. borotalensis* specimen was collected from Xinjiang, China (46°7′25.6″ N, 84°31′58″ E; Altitude: 840 m). *A. borotalensis* is not a legally protected species in China, despite the decline in the size of its population. Collection of the sample followed the wild plant protection regulations of China. The voucher specimen was deposited in the Herbarium of the Institute of Botany, Chinese Academy of Sciences (PE, Xian-Chun Zhang, zhangxc@ibcas.ac.cn), with collection number ZC-451.

## Methods

Total genomic DNA was extracted from approximately 100 mg of silica-dried leaf material using a modified CTAB method (Doyle and Doyle 1987). DNA extracts were fragmented to construct a 300 bp short-insert library and sequenced with 2 × 150 bp paired-end reads on DNBSEQ™ technology platforms at Beijing Genomics Institute (Shenzhen, China). The raw reads were filtered using Trimmomatic 0.35 (Bolger et al. 2014) to remove adapters and low-quality bases. Then, about 2.5 Gb of clean reads were assembled using GetOrganelle v. 1.7.1 (Jin et al. 2020). The chloroplast genomes were annotated with GeSeq (Tillich et al. 2017), before being manually adjusted with Geneious v. 9.1.7 (Kearse et al. 2012). Finally, the annotated chloroplast genomes were submitted to GenBank using Bankit (Accession number: ON964524), and Organellar Genome Draw (OGDRAW) (Lohse et al. 2007) was used to illustrate the circular genome map.

To investigate the phylogenetic position of *A. borotalensis*, we reconstructed its phylogenetic relationships based on 16 complete chloroplast genomes from 10 *Artemisia* species and used the closely related species *Ajania pacifica* (NC_050690) as an outgroup. The 17 complete chloroplast genomes (after removing one inverted repeat region) were aligned using the default parameters in MAFFT v. 7 (Katoh and Standley 2013). According to the Bayesian information criterion (BIC), the most appropriate substitution models, estimated using jModelTest2 (Darriba et al. 2012), were TVM + I + G for the complete chloroplast genome sequences. Maximum likelihood (ML) analyses were then conducted using RAxML-HPC v. 8 (Stamatakis 2014) with 1000 bootstrap replicates. The evaluation of the most appropriate substitution models and the construction of the phylogenetic tree were both carried out on the CIPRES Science Gateway portal (Miller et al. 2010).

## Results

**Figure 2.**
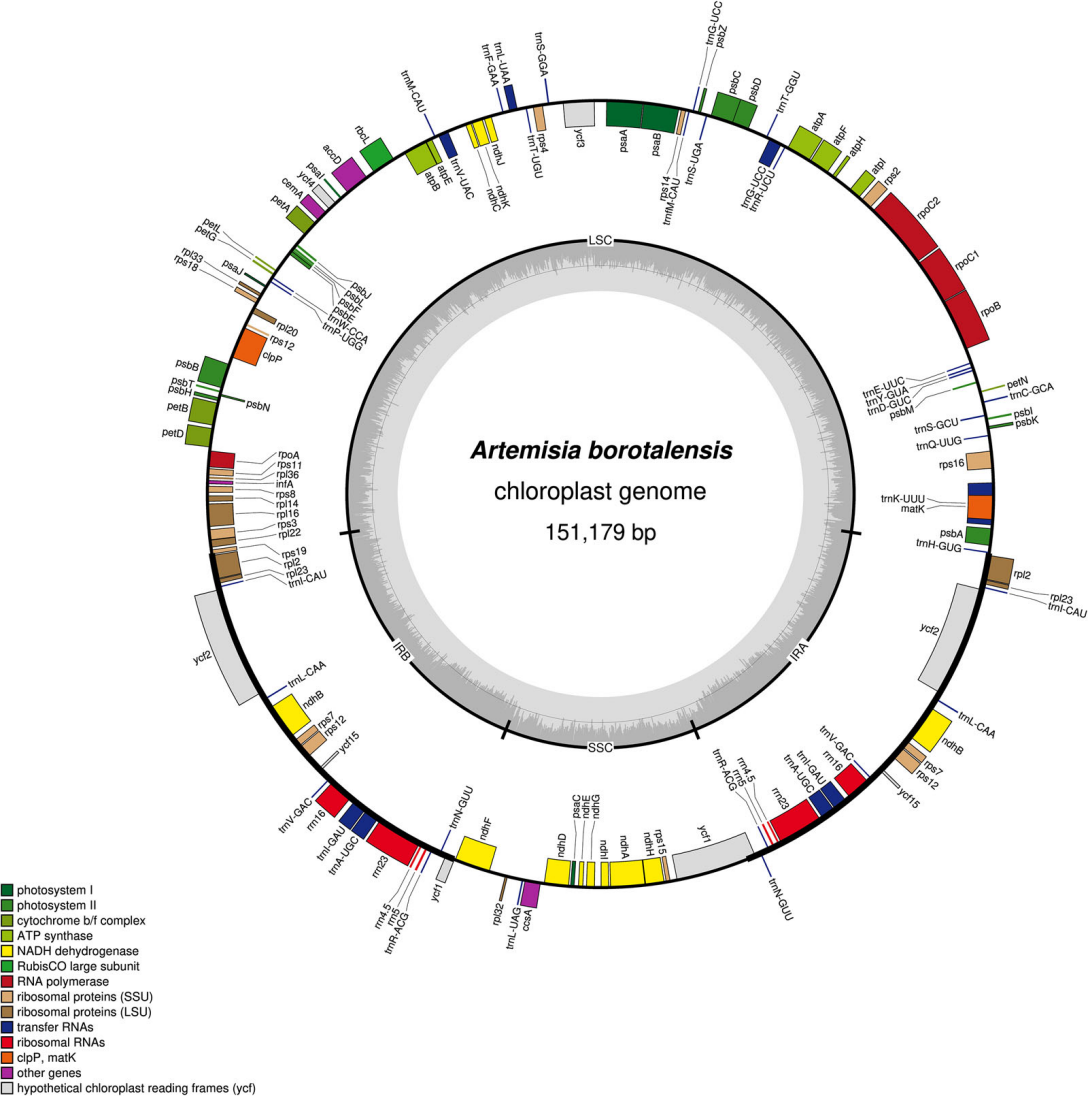
Circular chloroplast genome map for *Artemisia borotalensis* Poljakov. The colored bars indicate different functional gene groups. The thick lines of the large circle indicate the extent of the inverted repeat regions (IRa and IRb) that separate the genome into small single copy (SSC) and large single copy (LSC) regions, respectively. The darker gray columns in the inner circle correspond to the guanine-cytosine (GC) content, and the light gray columns to the adenosine-thymine (AT) content.

**Figure 3.**
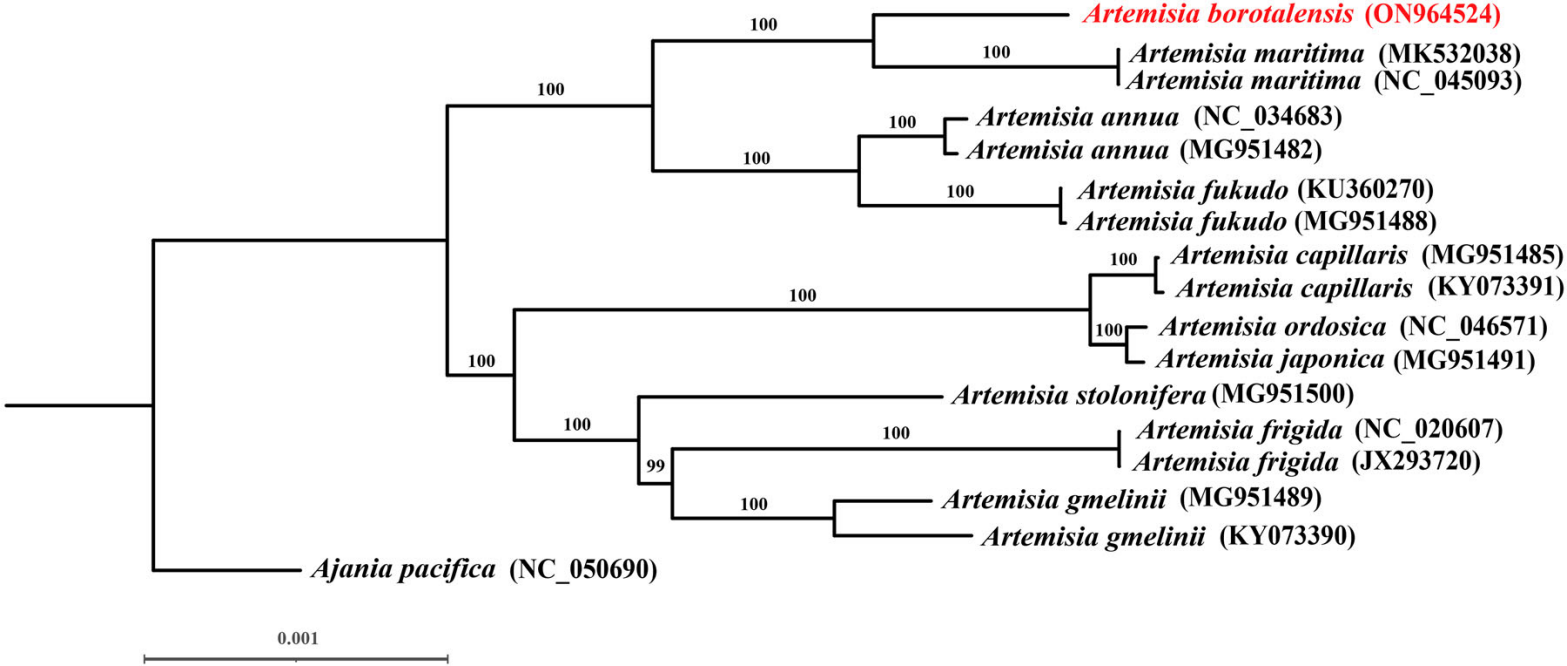
Phylogenetic trees inferred from maximum likelihood (ML) analyses based on 16 complete chloroplast genomes from 10 *Artemisia* species using *Ajania pacifica* as an outgroup with 1000 bootstraps replicates. The numbers above the branches indicate the bootstrap values. The species name colored in red represents our sequenced chloroplast genome (*A. borotalensis*). The following sequences were used: *Ajania pacifica* NC_050690 (Kim and Kim 2020), *Artemisia annua* MG951482 (Kim et al. 2020), *Artemisia annua* NC_034683, *Artemisia capillaris* KY073391 (Lee et al. 2022), *Artemisia capillaris* MG951485 (Kim et al. 2020), *Artemisia frigida* JX293720 (Liu et al. 2013), *Artemisia frigida* NC_020607, *Artemisia fukudo* KU360270 (Lee et al. 2016), *Artemisia fukudo* MG951488 (Kim et al. 2020), *Artemisia gmelinii* KY073390 (Lee et al. 2022), *Artemisia gmelinii* MG951489 (Kim et al. 2020), *Artemisia japonica* MG951491 (Kim et al. 2020), *Artemisia maritima* MK532038 (Shahzadi et al. 2020), *Artemisia maritima* NC_045093, *Artemisia ordosica* NC_046571 (Li et al. 2020), and *Artemisia stolonifera* MG951500 (Kim et al. 2020).

The sequenced chloroplast genome of *A. borotalensis* was found to possess a typical quadripartite structure, which consisted of large single copy (LSC), small single copy (SSC), and inverted repeat (IRa and IRb) regions (Figure 2). The complete chloroplast genome was 151,179 bp in length, of which the LSC, SSC, and IR were 82,862 bp, 18,377 bp and 24,970 bp in length, respectively. In total, 133 genes were identified, including 87 protein-coding genes, 37 transfer RNA genes, eight ribosomal RNA genes, and one pseudogene (*ycf1*). The overall GC content of the chloroplast genome was shown to be 37.4%. Comparison of the *A. borotalensis* chloroplast genome to previously published data revealed a high level of gene synteny with one publicly available genome data sets from *A. maritima* (Shahzadi et al. 2020). Furthermore, phylogenetic analysis showed that *A. borotalensis* and *A. maritima* clustered together as sister group (with 100% bootstrap support) to *A. annua* and *A. fukudo* clade (Figure 3).

## Discussion and conclusions

The chloroplast genome of *A. borotalensis* sequenced in this study was consistent with those of other previously published *Artemisia* taxa in terms of gene structure, number, and GC content (Kim et al. 2020), suggesting that the chloroplast genome of this taxon is relatively evolutionarily conserved. Moreover, the phylogenetic results confirmed the classification of *A. borotalensis* within the *Artemisia* subgenus *Seriphidium* based on its morphological traits, which has not been addressed in previous phylogenetic studies due to sampling limitations (Malik et al. 2017). In future, our findings will contribute to the development and conservation of the genetic resources of *Artemisia* and lay the foundation on which to develop a further understanding the phylogenetic relationships between members of this genus.

## Supplementary Material

Supplemental Material

## Data Availability

The authors have provided all the raw data, which has been all activated. The data that support the findings of this study are openly available in GenBank at https://www.ncbi.nlm.nih.gov/ under the accession number ON964524. The associated BioProject, SRA, and BioSample numbers are PRJNA857588, SRR20363929 and SAMN29630348 respectively.

## Regulation statement

All materials used in the study were collected in public areas of China and strictly follows the wild plant protection regulations of China. Voucher specimens were prepared and deposited at the Herbarium of the Institute of Botany, Chinese Academy of Sciences (PE).
