# Supplementary material for: The complete chloroplast genome of an endangered plant Artemisia borotalensis (Asteraceae) and phylogenetic analysis
Source: Mitochondrial DNA B Resour. 2023 Jan 15;8(1):145–8. doi: 10.1080/23802359.2022.2163599 (PMC9848251; doi:10.1080/23802359.2022.2163599)
Supplement: Supplemental Material [file TMDN_A_2163599_SM4935.docx]

**Supplementary Materials**


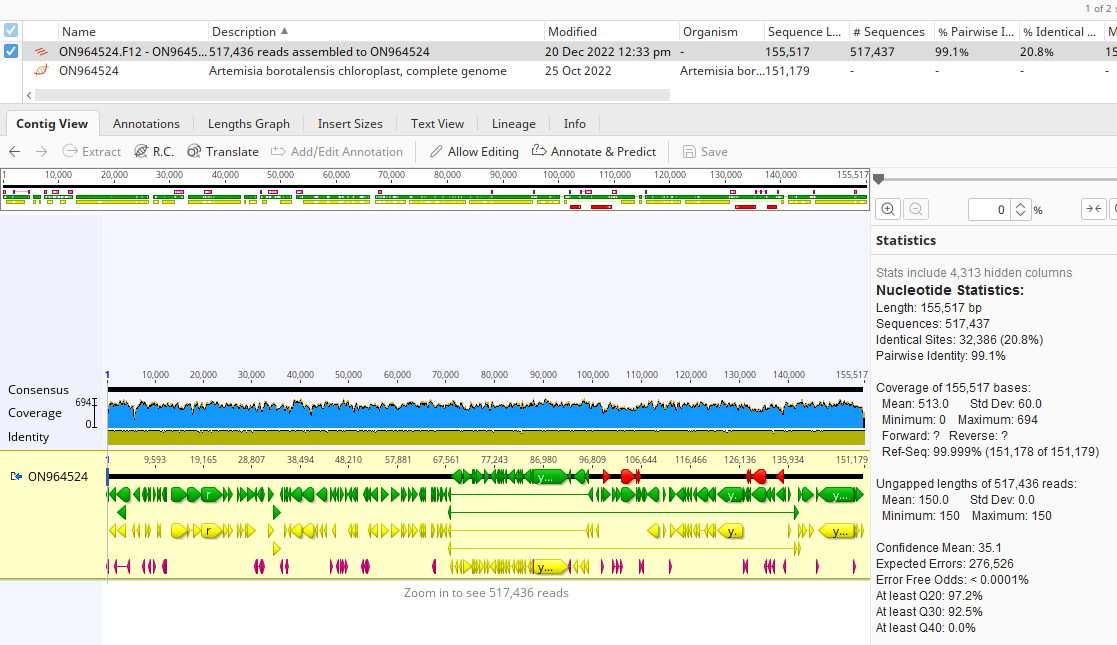


**Fig. S1:** Mapping of the quality of the chloroplast genome assembly of *Artemisia borotalensis* Poljakov (ON964524)

**Note：**In order to understand the sequencing and assembly quality, the coverage of each locus was counted using bowtie2 and samtools software, and then the files generated from the above results were imported into Geneious v. 9.1.7 (Kearse et al. 2012), and the final results are shown above. The average coverage is 513.

**
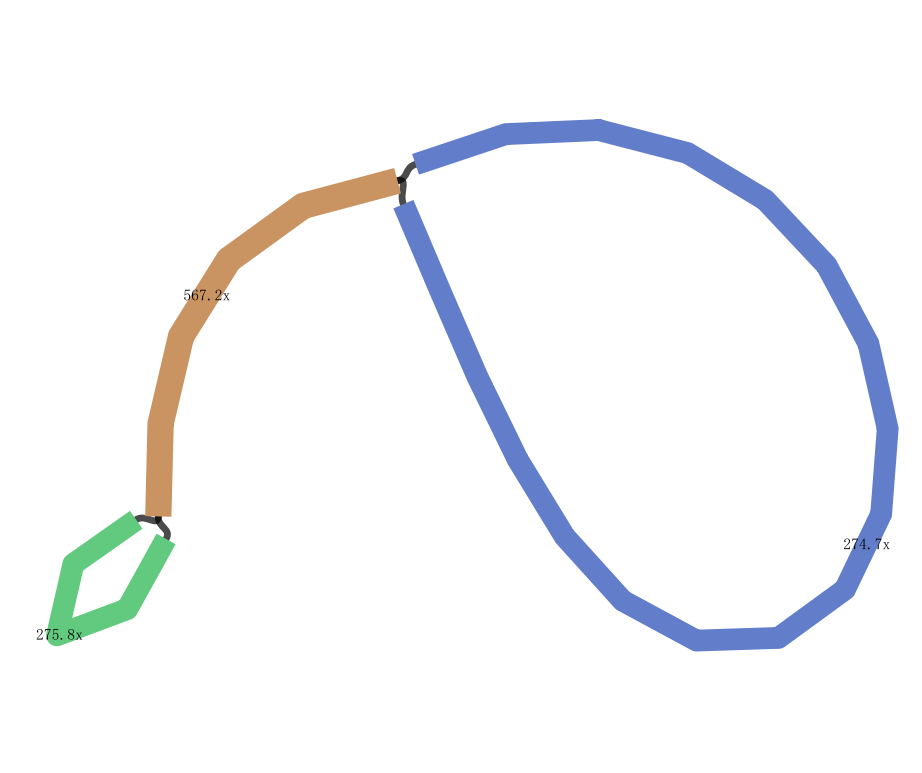
**

**Fig. S2:** Circular mapping of the chloroplast genome of *Artemisia borotalensis* Poljakov (ON964524)

**Note：**Complete circular assembly graph was checked by Bandage v. 0.8.1. The large fuchsia ring represents the large single copy region, the small fuchsia ring represents the small single copy region, and the thick light purple line represents the inverted repeat region. The three parts are well connected to represent the assembly of the chloroplast genome into a circle.
